# Chitosan-Based Electrochemical Sensors for Pharmaceuticals and Clinical Applications

**DOI:** 10.3390/polym15173539

**Published:** 2023-08-25

**Authors:** Alexandra Virginia Bounegru, Iulian Bounegru

**Affiliations:** 1 Department of Chemistry, Physics and Environment, Faculty of Sciences and Environment, “Dunărea de Jos” University of Galati, 47 Domnească Street, 800008 Galati, Romania; 2 Competences Centre: Interfaces-Tribocorrosion-Electrochemical Systems, “Dunărea de Jos” University of Galati, 47 Domnească Street, 800008 Galati, Romania; iulian.bounegru@ugal.ro; 3 Faculty of Medicine and Pharmacy, “Dunărea de Jos” University of Galati, 35 Al. I. Cuza Street, 800010 Galati, Romania

**Keywords:** sensors, drug, pharmaceutical analysis, carbon nanomaterials, metallic nanoparticles

## Abstract

Chitosan (CTS), a biocompatible and multifunctional material derived from chitin, has caught researchers’ attention in electrochemical detection due to its unique properties. This review paper provides a comprehensive overview of the recent progress and applications of CTS-based electrochemical sensors in the analysis of pharmaceutical products and other types of samples, with a particular focus on the detection of medicinal substances. The review covers studies and developments from 2003 to 2023, highlighting the remarkable properties of CTS, such as biocompatibility, chemical versatility, and large surface area, that make it an excellent candidate for sensor modification. Combining CTS with various nanomaterials significantly enhances the detection capabilities of electrochemical sensors. Various types of CTS-based sensors are analyzed, including those utilizing carbon nanomaterials, metallic nanoparticles, conducting polymers, and molecularly imprinted CTS. These sensors exhibit excellent sensitivity, selectivity, and stability, enabling the precise and reliable detection of medications. The manufacturing strategies used for the preparation of CTS-based sensors are described, the underlying detection mechanisms are elucidated, and the integration of CTS sensors with transducer systems is highlighted. The prospects of CTS-based electrochemical sensors are promising, with opportunities for miniaturization, simultaneous detection, and real-time monitoring applications.

## 1. Introduction

Detection and quantification of pharmaceutical substances in various matrices are essential for safeguarding human health and the environment, ensuring appropriate monitoring of medication therapies, and ensuring the quality of pharmaceutical products. The continuous development of new sensitive, selective, and cost-effective detection methods is necessary to meet current demands and ensure proper management of pharmaceutical substances in modern society.

Traditional analytical methods for pharmaceutical detection, such as liquid chromatography-mass spectrometry (LC-MS) [1], gas chromatography-mass spectrometry (GC-MS) [2], high-performance liquid chromatography (HPLC) [3], capillary electrophoresis (CE) [4], and immunoassays [5], are well-established and offer advantages such as high compound separation efficiency, sensitivity, and low sample volume requirements. While these methods have been instrumental in the field, they may also present challenges such as cost, time consumption, and the need for specialized equipment and skilled personnel, particularly in smaller or less-equipped laboratories [6,7].

Electrochemical methods, as explored in this review, present an alternative approach that complements these traditional techniques. With unique advantages such as affordability, rapid analysis, and minimal sample preparation, electrochemical methods are emerging as valuable tools in pharmaceutical detection. Rather than replacing other techniques, electrochemical methods offer additional options and flexibility, expanding the toolkit available to researchers and practitioners in the field.

Electrochemical sensors have emerged as promising approaches for pharmaceutical detection due to their high sensitivity, selectivity, and real-time monitoring capabilities, as well as the inherent advantages of miniaturization and portability of electrochemical instruments. These devices are based on the principle of converting a biochemical process into an electrical signal, typically involving the measurement of current or potential changes resulting from a redox reaction occurring on the sensor’s surface [8]. The miniaturization and portability of electrochemical instruments enable the development of compact and field-deployable sensors, expanding their applications and accessibility in various settings [9]. From our perspective, while these advancements are promising, there are still challenges to be addressed, such as reproducibility and long-term stability, which we will explore further in this review. Chitosan, a natural biopolymer derived from chitin, has been extensively explored for the development of electrochemical sensors due to its unique properties, such as biocompatibility, biodegradability, and large contact surface area [10]. Chitosan (CTS) can be easily functionalized and modified, making it an ideal material for electrochemical sensor development [11]. Combining CTS with other nanomaterials enhances electrochemical sensors’ sensitivity, selectivity, and stability. For example, carbon nanomaterials, such as graphene and carbon nanotubes, have been incorporated into CTS-based electrochemical sensors to improve the electron transfer rate and enhance electrocatalytic activity [12,13]. Metallic nanoparticles, such as gold and silver, along with CTS, can enhance the sensitivity and selectivity of sensors [14]. Additionally, conductive polymers like polyaniline and polypyrrole have been integrated with CTS to improve the stability and sensitivity of sensors [6,15,16].

This review article highlights recent progress in the development of CTS-based electrochemical sensors for pharmaceutical detection. This article explores in detail the natural origin and properties of CTS and provides a comprehensive introduction to the fundamental principles of electrochemical sensors. In the following sections, an exhaustive analysis of various types of CTS-based electrochemical sensors used for pharmaceutical detection is performed, including those involving the use of carbon nanomaterials, metallic nanoparticles, conductive polymers, and molecularly imprinted CTS. Finally, we discuss potential research directions in this field. Our article provides a critical and comprehensive analysis of innovative CTS-based electrochemical sensors, offering valuable insights for researchers and professionals in this rapidly expanding field.

This review explores electrochemical sensors based on CTS and describes their functionality in the detection of pharmaceutical substances. The detailed exploration of sensing principles provides a comprehensive understanding of the core technology behind these sensors. Key challenges and future directions are also discussed, highlighting the significant potential and areas for further research and development.

## 2. Natural Origin and Properties of CTS

Chitin, the second most abundant natural polymer after cellulose, is the source of the natural biopolymer CTS. Chitin is a structural polysaccharide found in the cell walls of fungi and certain algae, as well as in the exoskeleton of crustaceans, including shrimp, crabs, and lobster [17].

**Figure 1:**
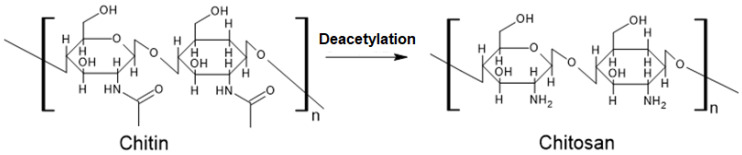
CTS formation from deacetylation of chitin.

CTS is produced by the deacetylation of chitin. This process involves treating chitin with alkaline solutions or specific enzymes that catalyze the removal of acetyl groups (-COCH_3_) from its structure. As a result of this reaction, chitin undergoes a chemical transformation into CTS. Once the acetyl groups are removed, a polymer with a higher degree of amino groups (-NH_2_) than chitin is obtained. One of the most important properties of this polymer is the presence of an amine group with a pKa value of 6.5 in the carbonic chain. This means that CTS has a positively charged surface, which imparts special properties and diverse applications in various fields [18]. The positive charge of CTS, along with its specific pKa value, gives it certain properties, such as the ability to interact with negatively charged molecules, such as nucleic acids or other biological molecules. This interaction can be exploited for drug delivery strategies, where CTS can act as a carrier and transporter for drugs or genetic material [19]. The structure of chitin and chitosan are presented in Figure 1.

Furthermore, CTS can be chemically or physically modified to introduce various functional groups, such as amino, carboxyl, or thiol groups, which can be used for the immobilization of biomolecules or for improving the electrochemical performance of sensors. Additionally, CTS can be modified by blending it with other polymers, such as polyvinyl alcohol or polyethylene glycol, to enhance its film-forming capability and mechanical properties [6].

Various studies have explored different aspects and applications in the field of CTS-based sensors. An extensive and important review by Suginta, Khunkaewla, and Schulte provides a comprehensive overview of (bio)sensors with chitin and CTS, detailing the advancements and applications of these materials in sensor technology [20]. Their work complements our findings and offers a broader perspective on the development and utilization of CTS-based sensors. The ease of modification and functionalization of CTS makes it a versatile material for the development of electrochemical sensors with improved sensitivity and selectivity [6]. In addition to its physical properties, the chemical characteristics of CTS, such as its ability to form stable complexes with metal ions, facilitate the incorporation of various types of nanomaterials, such as carbon nanotubes and metallic nanoparticles [21]. Its large contact surface area also enables it to function as an efficient matrix for the immobilization of biomolecules, such as enzymes and antibodies, in biosensor development [22]. Moreover, CTS has been found to exhibit antimicrobial activity [23], making it useful in the detection of pathogenic microorganisms [24,25]. Its ease of functionalization through chemical modification and versatility in various formulations further extend its possibilities for application in diverse fields [11,26]. The natural origin and properties of CTS make it an attractive material for the development of electrochemical sensors for pharmaceutical detection, among other applications. Its physical and chemical properties, including biocompatibility, biodisponibility, solubility, cationic nature, large contact surface area, antimicrobial activity, and functional ability, offer advantages in the development of sensors with improved performance and cost-effectiveness [27].

## 3. Principles of Electrochemical Sensors

Electrochemical sensors have emerged as vital tools in various fields, including pharmaceutical detection. The sensing principles of these sensors are fundamental to understanding their functionality and applications. In this section, we will explore the underlying principles that govern the operation of different types of electrochemical sensors based on CTS.

Electrochemical sensors have several advantages that make them a popular choice for detecting and measuring various chemical species. Some of the key advantages of these sensors include their high sensitivity, selectivity, and fast response time.

Recent research has highlighted the potential of electrochemical sensors for a wide range of applications. For example, a study published in the Journal of Hazardous Materials found that electrochemical sensors can be used to detect and quantify heavy metal ions in water samples [28]. Another study, published in Materials Today Chemistry, demonstrated the use of electrochemical sensors for detecting volatile organic compounds in air samples [29].

In addition to their high sensitivity and selectivity, electrochemical sensors are also known for their low cost and ease of use. A review published in Environmental Research highlighted the potential of these sensors for environmental monitoring, particularly in developing countries where resources are limited [30].

Furthermore, advances in materials science and nanotechnology have led to the development of new types of electrochemical sensors with improved performance. A recent report in Sensors and Actuators Reports described the use of nanomaterials to enhance the sensitivity and selectivity of electrochemical sensors [31].

The fundamental principle of any electrochemical sensor, as defined by the latest IUPAC terminologies [32], involves the recognition of the analyte through its interaction with the active layer of the material it is composed of, followed by the transduction of the signal to the recording equipment. Electrochemical sensors for analytes dissolved in solution generally consist of electrochemical cells placed in a specific arrangement with the working electrode’s active material (the sensing unit), the analyte, the reaction medium, and the set of electrodes used.

Electrolytic processes occur at the interface, where the polarization of the electrodes (cathode and anode) induces an electrochemical reaction. Specifically, these sensors exhibit a change in current or potential in response to a redox reaction occurring at the sensor’s surface. In a conventional two-electrode electrolytic cell, as described by IUPAC, there is a current source, a voltmeter, an anode, and a cathode. Sensors can primarily be classified into two types: potentiometric and amperometric, following the IUPAC definitions. Amperometric sensors measure changes in the current generated by an electrochemical reaction at the sensor’s surface, while potentiometric sensors quantify potential changes [33].

**Figure 2:**
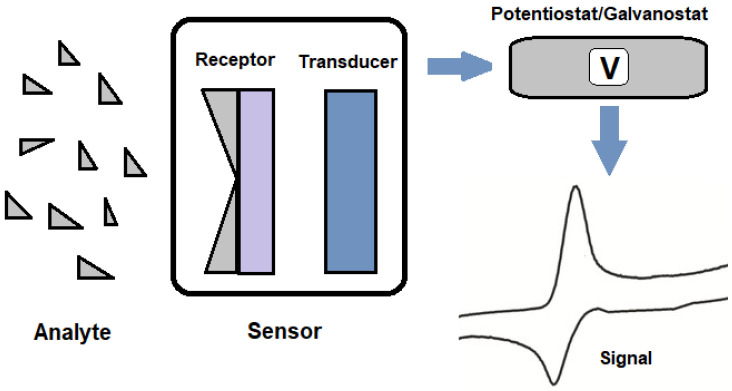
Schematic of an electrochemical sensor.

The schematic representation of an electrochemical sensor is shown in Figure 2.

The working efficiency of an electrochemical sensor stems from a complex interaction of several factors, including the type of electrodes, the nature of the electroactive species, the composition of the electrolyte solution, and specific design parameters of the sensor, all according to IUPAC guidelines. Regarding the electrode categories, three essential classes stand out: working electrodes, reference electrodes, and auxiliary electrodes [34]. At the working electrodes, the essential electrochemical reaction for the detection mechanism takes place, making them the critical component of the sensor. On the other hand, reference electrodes provide a stable reference potential to the working electrode, ensuring the accuracy and reproducibility of measurements. Auxiliary electrodes facilitate the flow of electrons with the working electrode, enabling signal generation and detection, while reference electrodes provide a stable reference potential to the working electrode, ensuring the accuracy and reproducibility of measurements [33,35].

The selection of the material for the working electrodes influences the sensitivity, selectivity, and stability of the sensor. Typical materials for working electrodes include noble metals and conducting polymers, as recognized by IUPAC [13,32,36]. The sensitivity and selectivity of electrochemical sensors can be enhanced through modifications to the active surface. Functionalizing the surface with molecules such as CTS can provide a stable and biocompatible platform for biomolecule immobilization [37]. CTS can act as a weak reducing agent in the synthesis of metallic nanoparticles, such as gold nanoparticles. These applications start with the presence of free amino groups and hydroxyls throughout the CTS chain. Additionally, by incorporating nanomaterials such as carbon nanotubes, metallic nanoparticles, and quantum dots, the electrochemical performance can be improved [38].

Potentiometric sensors operate on the principle of measuring the potential difference between a working electrode and a reference electrode. The potential difference is directly related to the concentration of the target analyte. In CTS-based potentiometric sensors, the biocompatibility and stability of CTS enhance the sensor’s selectivity and sensitivity.

Amperometric sensors measure the current resulting from the electrochemical oxidation or reduction in an analyte. CTS can be used as a matrix to immobilize enzymes or other recognition elements, providing a stable environment that allows for accurate current measurement correlated to the analyte concentration.

Impedimetric sensors work by measuring the impedance or resistance to the flow of an alternating current in the presence of the target analyte. The interaction between CTS and the analyte can cause changes in impedance, providing a means to detect specific substances [13,32,36].

While CTS-based electrochemical sensors offer numerous advantages, understanding their limitations is essential. Challenges include enhancing specificity in complex sample matrices, scalability, commercialization, and integration with other technologies.

However, the use of electrochemical sensors is not without challenges. While progress in sensor development is evident, maintaining a balance between advanced sensor design and practical applicability remains a challenge. The robustness, reliability, and durability of these sensors in real-world applications require additional attention. The integration of biomaterials such as CTS and other types of nanomaterials represents a promising avenue, but these modifications introduce new complexities in the sensor design and functionality that need to be carefully addressed to fully exploit the potential of electrochemical sensors, in line with the latest IUPAC standards. The future of CTS-based electrochemical sensors lies in investigating innovative methods, applications in emerging fields, sustainability considerations, and encouraging interdisciplinary collaboration and standardization.

## 4. Electrochemical Sensors Based on CTS for Pharmaceutical Detection

Electrochemical sensors based on CTS have established a significant role in various industries, including the chemical industry, the food industry, environmental science, and the pharmaceutical industry [15,39]. While the possibilities of functionalizing CTS, its biocompatibility, and its relative accessibility are promising, challenges persist in improving the stability and reproducibility of CTS-based sensors and establishing reliable methods for large-scale manufacturing. These issues highlight the need for critical analysis of CTS-based sensor construction strategies to chart future research and innovation directions in this field.

CTS-based electrochemical sensors have demonstrated evident adaptability in a variety of applications. They have been successfully used in the detection of antiallergic substances such as phenylephrine, chlorpheniramine maleate, dextromethorphan, and cetirizine due to the high prevalence of allergic diseases [40,41]. Furthermore, these sensors are valuable in the detection of neoplastic markers for early disease diagnosis [42,43,44], such as aptasensors for detecting human epidermal growth factor receptor 2 proteins, a critical prognostic biomarker for breast cancer [43], or the nanosensor for detecting sarcosine, a biomarker for prostate cancer, using composite films of carbon nanotubes and CTS [44]. Moreover, the literature reports electrochemical sensors based on CTS for the detection of a wide range of pharmaceutical substances with various indications, such as paracetamol [45], diclofenac [46], clindamycin [47], metformin [48], propranolol [49], and fluoxetine [50], as well as a series of metabolites present in biological fluids, such as cholesterol [51], uric acid [52], xanthine, hypoxanthine, p53 protein, and purines, considered potential biomarkers [6]. CTS-based sensors have also been utilized for the detection of narcotics and their metabolites, such as cocaine [53], morphine [54], and methamphetamine [55]. This is a global concern, considering the misuse and dependence on such substances, making their detection in biological samples and forensic investigations a priority [56].

The following sections will present in detail how CTS-based sensors can be functionalized with different categories of nanomaterials for the detection of pharmaceutical substances in various types of samples.

### 4.1. Electrochemical Sensors Based on CTS and Carbon Nanomaterials

The incorporation of carbon nanomaterials such as graphene, carbon nanotubes, and carbon nanofibers into CTS-based structures presents significant potential in sensor technology, particularly in the pharmaceutical field. Carbon-based nanomaterials contribute to remarkable conductivity and substantial contact surface area, enabling the detection of pharmaceutical substances even at nanomolar concentrations, and making them valuable components in the design of electrochemical sensors based on CTS [57,58].

Carbon paste electrodes are among the most widely used in electrochemical analysis due to their excellent properties [59]. These electrodes are fabricated by mixing carbon graphite powder with mineral oil or silicon to obtain a consistent and malleable mass. Carbon paste electrodes provide a large surface area for interaction between the analyte and the electrode, allowing for efficient electron transfer and rapid electrochemical reactions. Modification with CTS, for example, can enhance the sensitivity and selectivity of the electrodes, enabling more precise detection and quantification of the target analytes [60,61].

Such an electrode was developed and characterized for the quantification of paracetamol. The modified mixture was obtained by incorporating carbon graphite powder into a pre-prepared CTS gel. Compared with unmodified electrodes, it was observed that the anodic current intensity specific to paracetamol oxidation was significantly higher, indicating increased method sensitivity. Experimental parameters were optimized, and the method demonstrated good repeatability, while selectivity was evaluated by testing interferences with other substances present in the solution (ibuprofen, ascorbic acid, and uric acid). The proposed method was successfully applied to the determination of paracetamol in natural water samples, tablets, and urine [62].

In particular, sensors incorporating carbon nanotubes or graphene into a CTS matrix have demonstrated exceptional sensitivity and selectivity in detecting a wide range of drugs.

A representative example is a composite sensor based on carbon nanotubes and CTS designed for the detection of tetracycline. Tetracycline, an antibiotic commonly used in both veterinary and human medicine, often contaminates the environment through agricultural runoff and waste disposal. This particular sensor demonstrates excellent sensitivity and selectivity for detecting tetracycline, down to a detection limit of 0.11 ng/mL, due to the stable and biocompatible surface provided by CTS for immobilizing tetracycline antibodies [63].

A simple and efficient method was applied by Biljana Nigovic *et al.* for constructing a CTS-based sensor functionalized with amino groups on carbon nanotubes for the simultaneous determination of mesalazine and folic acid. Before modification, the glassy carbon electrode (GCE) was cleaned with alumina paste and water. The functionalized multi-walled carbon nanotubes (MWCNTs) were dispersed in a CTS and Nafion solution and subsequently uniformly applied onto the pre-polished GCE surface. The anodic signals of both analytes were enhanced due to their strong adsorption capacity and electrochemical properties. The sensor was tested for the quantification of both pharmaceutical substances in serum samples and pharmaceutical products, demonstrating its utility in monitoring the treatment of patients with inflammatory bowel disease [64].

In 2017, a nanosensor based on nitrogen-doped carbon nanotubes (N-CNTs) and CTS, supported by a GCE (N-CNTs-CHIT/GCE), was developed for the amperometric characterization of rabeprazole, a proton pump inhibitor used for the treatment of gastric ulcers. In this case, N-CNTs are synthesized using a Fe@MgO catalyst and melamine formaldehyde as a powder. After synthesis, the final product (N-CNTs) undergoes additional steps of thermal treatment and removal of metal precursors and metal oxides. The N-CNTs solution is then mixed with the CTS solution, and the resulting suspension is applied to the pre-polished GCE surface. This results in an N-CNTs-CHIT/GCE nanohybrid, which exhibits a conductive network and a large electrocatalytic surface. N-CNTs-CHIT/GCE demonstrates superior electrocatalytic performance in the oxidation of rabeprazole and provides a remarkable amperometric response in a solubilized system with sodium dodecyl sulfate, minimizing the use of hazardous organic solvents during electroanalysis [65].

**Figure 3:**
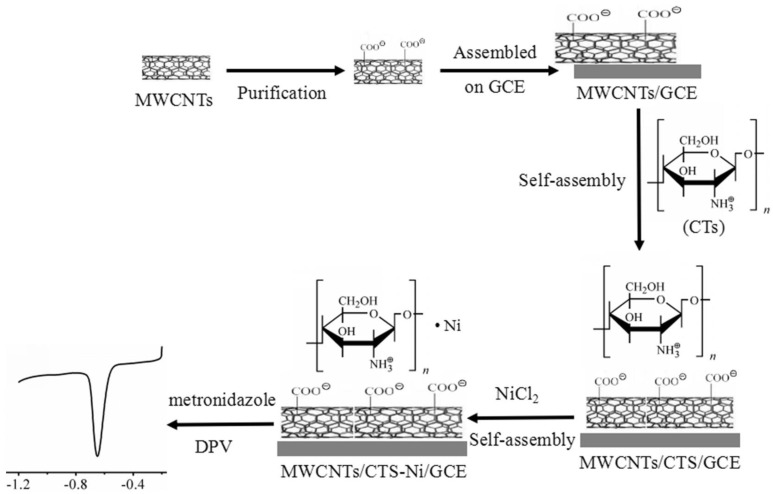
Schematic diagram of the self-assembly process of MWCNTs/CTS-Ni/GCE [66].

By using the self-assembly technique, another electrochemical sensor based on MWCNTs and complex CTS-nickel (MWCNTs/CTS-Ni) was fabricated for sensitive metronidazole detection. In the first step, the surface of the GCE was polished and coated with an MWCNTs suspension, followed by drying at room temperature. In the second step, these electrodes were immersed in a CTS solution for 30 min, allowing for the self-assembly of CTS on the MWCNT’s surface. The electrodes were then rinsed and immersed in a nickel solution for 30 min, facilitating the self-assembly of nickel ions on the CTS surface. The electroanalytical performance of the proposed modified electrode, MWCNTs/CTS-Ni, was thoroughly evaluated, demonstrating excellent electrocatalytic activity toward metronidazole. The proposed sensor showed a linear response to metronidazole concentrations ranging from 0.1 to 150 μmol L^−1^, with a low detection limit of 0.025 μmol L^−1^. Moreover, the sensor exhibited good selectivity, reproducibility, and stability. The proposed method can be successfully applied to the sensitive determination of metronidazole in pharmaceutical and biological samples [66]. A schematic diagram of the process is represented in Figure 3. 

The same technique was used to construct an electrochemical sensor for paracetamol based on MWCNTs and a CTS-copper complex (MWCNTs/CTS-Cu). The GCE was polished with alumina and nitric acid solutions, and then the carbon nanotube suspension was dispersed onto the active surface of the electrode. After drying, the sensor was immersed in a CTS solution. Positively charged CTS self-assembles on MWCNTs with carboxylic functional groups through electrostatic interactions. Subsequently, the electrode was immersed in a copper solution, leading to the self-assembly of Cu^2+^ on CTS through complexation actions. The result was a modified sensor, MWCNTs/CTS-Cu-modified GCE, which exhibited excellent electrocatalytic activity for the oxidation of paracetamol and facilitated electron transfer between the electrode and paracetamol. Under optimal experimental conditions, the maximum differential pulse current was directly proportional to the concentration of paracetamol in the range of 0.1 to 200 μmol L^−1^, with a detection limit of 0.024 μmol L^−1^. The sensitivity of the sensor was 0.603 A/mol L^−1^. Moreover, the proposed sensor showed high selectivity for paracetamol in the presence of ascorbic acid and dopamine. Additionally, the proposed electrode demonstrated good reproducibility and stability and was applied for the determination of paracetamol in tablets and human serum [67].

The combination of CTS with graphene, due to their unique electrical properties and significant surface area, has been extensively used in the development of electrochemical sensors. For example, Feng *et al*. developed an electrochemical sensor using a CTS/graphene oxide nanocomposite for the detection of melamine in milk samples, demonstrating the practical utility of these nanocomposites [68].

A new nanocomposite of reduced graphene oxide (RGO), carbon black (CB), and CTS was used to modify a GCE named RGO-CB-CTS/GCE for the simultaneous determination of dopamine and paracetamol. The sensor modification strategy allowed for the preparation of a stable dispersion and an efficient electroactive layer. By employing cyclic voltammetry and the Nicholson method, an electron transfer rate constant (k_0_) of 5.6 × 10^−3^ cm s^−1^ was obtained. The developed sensor exhibited excellent selectivity and sensitive detection limits for both analytes. This new electrode modification method is simple, cost-effective, and provides a rapid and precise response for the simultaneous analysis of dopamine and paracetamol [69].

When two-dimensional graphene is transformed into zero-dimensional graphene quantum dots (GQDs) with sizes smaller than 10 nm, they acquire remarkable electrochemical properties and adjustable luminescent behavior [70]. Essentially, GQDs represent small fragments of graphene where electronic transport is confined in all three dimensions. GQDs offer an intriguing platform for nanotechnology research and development, showing promising prospects in the field of nanoscience and technology [71].

Thus, Javad Tashkhourian *et al*. used GQDs and CTS as stabilizers to modify a carbon paste electrode for the detection of epinephrine. CTS was employed to maintain the solubility of GQDs in an aqueous solution. Consequently, a nanocomposite was prepared where GQDs were surrounded by CTS, promoting electrostatic interaction between the carboxylic groups with negative charges and the amino groups in CTS. This combination of GQDs and CTS, along with the conjugated π-π bond, proved to be a stable environment and an excellent tool for the detection of epinephrine. The dependence between the maximum current and the concentrations of epinephrine on the modified electrode is linear, with a detection limit of 0.3 nM. This discovery represents a promising nanoscale platform and opens new possibilities in the design of advanced electrochemical sensors [72].

In Table 1 we listed the main electrochemical sensors based on CTS and carbon nanomaterial. 

Based on the extensive literature, the distinctive characteristics of carbon nanomaterials, specifically their expansive surface area and augmented electrochemical attributes, when synergistically integrated with the biocompatibility and stability inherent to CTS, culminate in the fabrication of sensors that exhibit unparalleled sensitivity and selectivity. For example, the detection of paracetamol, often from pharmaceutical tablets, is more frequently pursued, with square wave voltammetry being the preferred voltammetric technique. However, although these sensors have shown great potential in the laboratory, their practical implementation can still be challenging. Aspects such as stability and the reproducibility of sensor responses merit continuous exploration. Additionally, the development of these sensors for detecting a broader range of pharmaceutical substances, especially those with high toxicity or low solubility, requires further research.

Furthermore, it is important to evaluate these sensors from a commercial perspective. Field applicability often demands strict operating conditions, long-term sensor stability, and a high degree of precision—parameters that still need improvement. Moreover, the scalability of these sensors must be considered, involving aspects related to manufacturing processes, cost-effectiveness, and adaptability to various detection platforms.

### 4.2. Electrochemical Sensors Based on CTS and Metallic Nanoparticles

The combination of CTS with metallic nanoparticles represents a promising approach to the development of electrochemical sensors. This synergy opens new perspectives in various fields, including biotechnology, biomedicine, and environmental monitoring. This section explores different applications, techniques, and materials used in this innovative field.

Metallic nanoparticles such as gold, silver, or copper exhibit a large surface area and high chemical reactivity. By associating CTS with metallic nanoparticles, composite materials with improved properties, including stability, reactivity, and biocompatibility, can be obtained.

For example, Maryam Ehsani *et al.* applied cyclic voltammetry for the electrochemical deposition of silver nanoparticles (AgNPs) on the surface of a glassy carbon electrode (GCE), followed by the polymerization of a CTS film. This modification process enhanced the electrocatalytic behavior of the electrode, enabling precise determination of doxorubicin (DOX) in cellular lysate and plasma samples [75].

Different techniques have been employed for the detection of various substances. In the case of aspirin detection, a screen-printed electrode (SPCE) was modified with a hydrogel consisting of a gold nanoparticle and CTS solution and successfully used for the detection of aspirin in urine, saliva, and pharmaceutical tablets [76].

Metal oxides have the advantage of combining the specific properties of metals with those of oxides, providing them with a range of unique characteristics for sensor modification. Due to their crystalline structure and precise chemical composition, metal oxides can be optimized to possess specific semiconductor, catalytic, or sensorial properties, making them highly useful in the field of electrochemical sensor development.

**Figure 4:**
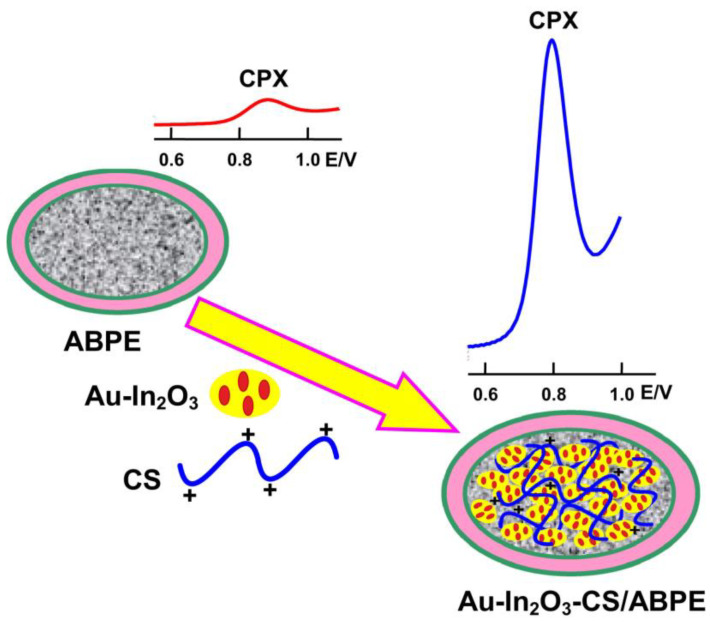
Schematic diagram of the CPX detection at ABPE and at the Au-In_2_O_3_-CS/ABPE [77].

For example, in 2018, gold nanoparticles were deposited on the surface of In_2_O_3_, which, together with CTS, modified a carbon paste electrode for the detection of ciclopirox, an antifungal agent commonly used in pharmaceutical preparations and as an anti-seborrheic agent in cosmetics. Gold nanoparticles enhance the conversion of oxygen species and the reaction rate between oxygen species and target molecules, ultimately improving detection properties. Gold-deposited In_2_O_3_ nanoparticles and the Au- In_2_O_3_ nanocomposite were synthesized through the hydrothermal method. The modified electrode, Au- In_2_O_3_-CS/ABPE, was obtained by adding the Au-In_2_O_3_ nanocomposite and CTS to the carbon paste mixed with paraffin oil (Figure 4). The paste was then compacted into a Teflon tube.

In 2018, gold nanoparticles were deposited on the surface of In_2_O_3_, which, together with CTS, modified a carbon paste electrode for the detection of ciclopirox, an antifungal agent [77]. Another study proposed the use of CTS and ZrO_2_ nanoparticles for the detection of rifampicin, an antitubercular drug [78].

By associating metallic nanoparticles with carbon nanomaterials, a strong synergy is achieved between these components, maximizing the benefits of both. Metallic nanoparticles can be effectively anchored or encapsulated in the carbon matrix, resulting in a large contact surface area and strong interactions. This interaction significantly improves catalytic performance, electrical conductivity, and sensor sensitivity. For example, by covalently functionalizing CTS with MWCNTs, the strength and mechanical performance of the resulting nanocomposite are enhanced. Thus, the proposed carbon paste electrode by Nehad A. Abdallah *et al.* combines titanium nanomaterials deposited on MWCNTs with the covalent attachment of CTS on their surface. This configuration allowed for sensitive and selective detection of pazufloxacin in various solid samples and human plasma [79]. By associating metallic nanoparticles with carbon nanomaterials, a strong synergy is achieved. For example, a nanocomposite prepared from silver nanoparticles, CTS, and carbon nanotubes was used for the detection of clopidogrel, an antiplatelet agent [80].

**Figure 5:**
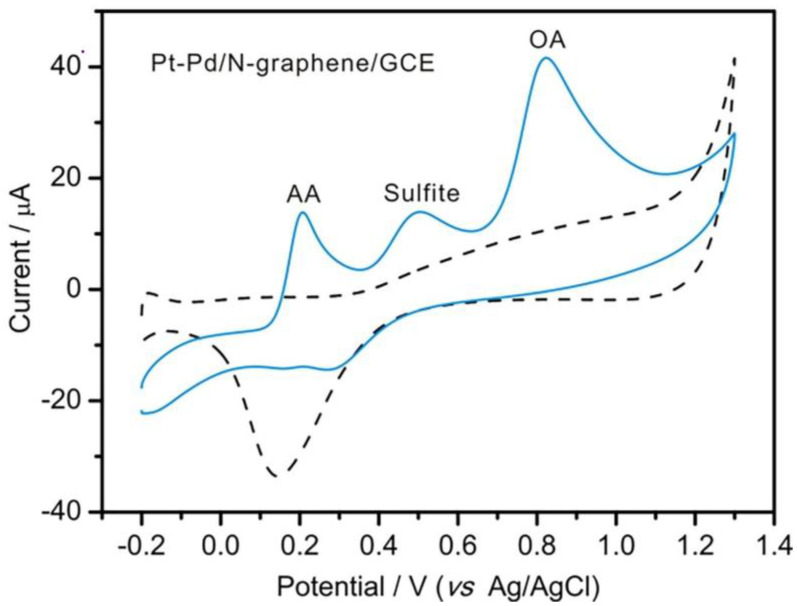
CVs of a mixture solution containing ascorbic acid (AA), sulfite, and oxalic acid (OA) were recorded at Pt-Pd NPs/N-Gra/GCE. Shown in solid and dashed traces were the mixture analytes (1 mM each) and the blank electrolytes (50 mM PBS, pH 4.0), respectively. Scan rate: 50 mV/s [81].

Developing a sensitive analytical method for the simultaneous detection of multiple analytes is a challenge. Xiaoli Luo and colleagues overcame this challenge by developing an innovative sensor for the simultaneous detection of ascorbic acid, sulfite, and oxalic acid [81]. Sulfite and oxalic acid, which can cause allergic reactions, have cytotoxic effects, and contribute to kidney stone formation, are considered undesirable substances in pharmaceutical formulations. Thus, developing a sensitive detection method for these substances is essential for ensuring rigorous monitoring and optimal clinical drug quality. Bimetallic nanoparticles were synthesized through one-step electrodeposition at a constant potential, eliminating the need for reducing agents, stabilizers, or surfactants. The sensor demonstrated remarkable sensitivity and selectivity in the simultaneous detection of these compounds. Figure 5 shows the CVs of a mixture solution containing ascorbic acid (AA), sulfite, and oxalic acid (OA).

The proposed electrochemical method allowed for rapid and precise measurement of these critical compounds in clinical medications without the need for chromatographic separation methods [81].

Graphene oxide, being a hydrophilic material with a hexagonal structure consisting of sp^2^ and sp^3^ hybridized carbon atoms and functional groups (hydroxyl, epoxy, carboxyl, and carbonyl) located on the basal and edge planes, exhibits an affinity for metallic nanoparticles, resulting in a promising nanocomposite with significantly improved electrocatalytic properties [82]. Therefore, Ademar Wong *et al.* proposed an electrochemical method for the detection of clindamycin using a GCE modified with graphene oxide and gold nanoparticles in a CTS film cross-linked with epichlorohydrin. The method demonstrates an extended linear concentration range and a low detection limit, successfully applied in the quantification of clindamycin in various sample types, including pharmaceutical samples [47]. The sensor exhibits high stability. On the other hand, for dopamine detection, a carbon paste containing a nanocomposite of ZrO_2_, graphene, and CTS was chosen, which was subsequently introduced into a glass tube, thus constructing a sensitive and selective sensor toward ascorbic acid and uric acid [83]. Table 2 presents several electrochemical sensors based on CTS and metallic nanoparticles, along with the electrochemical technique used, the target analyte, and the corresponding detection limits

The integration of CTS with metallic nanoparticles has led to significant advancements in electrochemical sensors. These sensors have found diverse applications in pharmaceuticals, offering improved properties such as sensitivity, selectivity, and stability. Future research may further explore the potential of these composite materials in various analytical applications.

### 4.3. Electrochemical Sensors Based on CTS and Conducting Polymers

CTS possesses film-forming capacity and good mechanical strength but suffers from poor electrical conductivity. To overcome this limitation, CTS can be combined with conducting polymers such as polyaniline and polypyrrole, enhancing charge transfer, sensitivity, and selectivity of electrochemical sensors [7,87,88].

CTS’s unique ability to form stable films on solid substrates, such as glassy carbon, has been widely recognized. Even after drying the film, it exhibits remarkable stability, remaining intact when submerged in acidic solutions. This stability is attributed to the strong intermolecular interactions within the CTS structure, including hydrogen bonding and electrostatic interactions with the substrate. Such resilience makes CTS an attractive material for various applications, including electrochemical sensors, where the stability of the film can enhance the sensor’s performance, reliability, and lifespan [58]. Further studies exploring the mechanisms behind this stability and potential enhancements through modifications or combinations with other materials could open new avenues for innovation in sensor design and other fields [60,89,90,91].

One of the common techniques for preparing conducting polymer composites based on CTS is in situ polymerization, which involves polymerizing monomers in the presence of CTS. For example, Zad *et al.* [92] used this technique to synthesize a CTS-polyaniline-Fe_3_O_4_-Ni-Pd composite for the detection of fluconazole. Fluconazole is an antifungal drug widely used in clinical practice that can cause adverse effects if overdosed or abused. In the first step, Fe_3_O_4_@SiO_2_ nanoparticles were obtained by coating Fe3O4 nanoparticles with silicon. Furthermore, oxidative polymerization of aniline was carried out on the surface of Fe_3_O_4_@SiO_2_ to obtain the Fe_3_O_4_@SiO_2_@PA composite. In the next step, Ni@Pd was immobilized onto the Fe_3_O_4_@SiO2@PA composite by NaBH4 reduction. Subsequently, carbon paste electrodes with ionic liquid were modified by adding Fe_3_O_4_@SiO_2_@PA-Ni@Pd nanoparticles and a CTS solution. The modified electrodes exhibited rapid electron transfer and significant electrocatalytic activity in the detection of fluconazole. This new nanoscale platform also holds potential for the detection of other electroactive drugs [92].

Another technique for preparing CTS-based polymer composites is drop casting, which involves depositing a solution or suspension containing the composite onto a substrate. This technique was also used to modify a GCE with graphene nanosheets, CTS, and poly(amidoamine) dendrimer to investigate the electrochemical behavior of rutin in a pH 6.0 buffer solution. The results showed that the modified electrode exhibited enhanced electrochemical activity towards rutin, with significantly higher oxidation current intensity compared with other electrodes. Multiple parameters, such as the amount of immobilized graphene and dendrimer, pH, scan rate, accumulation time, and potential, were optimized. Kinetic parameters were calculated, and a linear relationship between the maximum oxidation current and rutin concentration in the range of 0.001–2.0 μmol L^−1^ was obtained, with a correlation coefficient close to 1. The detection limit was 0.6 nmol L^−1^. The modified electrode demonstrated satisfactory selectivity and reproducibility and was successfully used for the determination of rutin in pharmaceutical preparations and biological samples, with recoveries exceeding 97% [93].

Electrodeposition is another frequently used and efficient technique for coating electrodes with polymer composites. In the study by Ali M.A. Abdul Amir AL-Mokaram, a one-step electrodeposition method was developed to obtain Polypyrrole-CTS- Fe_3_O_4_ (Ppy-CS-Fe_3_O_4_NP/ITO) nanocomposite films. This method allowed for the dispersion of Fe_3_O_4_ nanoparticles in CTS solution (Cs) and the formation of a viscous solution with uniform dispersion. Furthermore, a solution of pyrrole and p-TS was added, and the Ppy-CS- Fe_3_O_4_ nanocomposite film was electrodeposited on an indium tin oxide (ITO) glass electrode using cyclic voltammetry. This approach led to the formation of a modified Ppy-CS- Fe_3_O_4_NP/ITO electrode with a rapid amperometric response, high selectivity, and non-enzymatic glucose detection capability, exhibiting an extended linear range and low detection limit [94].

**Figure 6:**
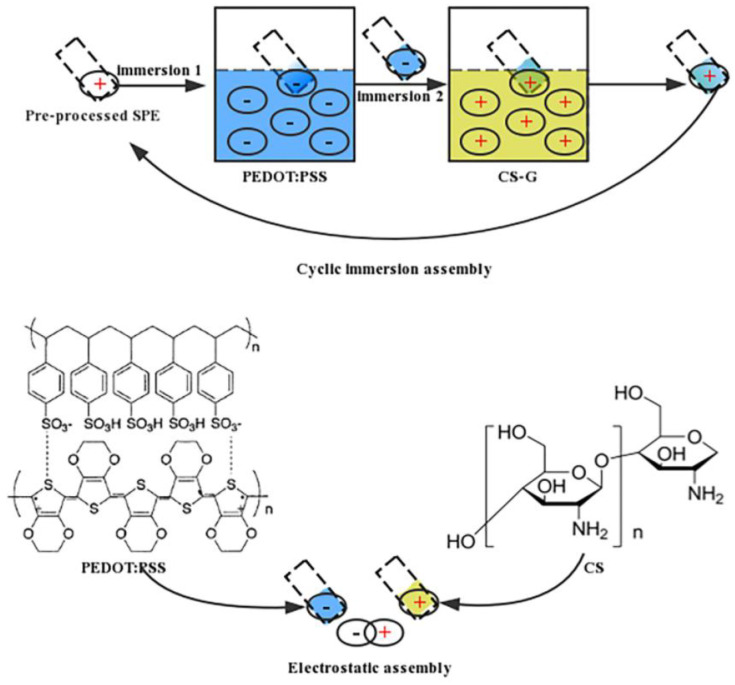
The process of layer-by-layer electrostatic self-assembly [95].

On the other hand, the use of smartphones in electrochemical detection applications represents a promising and innovative technology in the field of point-of-care testing (POCT). A relevant example of this technique is the portable smartphone-based electrochemical testing system developed in the research by Xiaoyan Shen *et al.* [95]. This device enables the detection and analysis of biomarkers through a smartphone, allowing for real-time monitoring of specific substance concentrations. To achieve the modification, several steps were followed in the preparation of solutions and their application to SPCEs. Before modification, the SPCEs were prepared by washing and sonication. To ensure better adhesion of the modification materials, reference electrodes, and counter electrodes were temporarily covered with hydrophobic double-sided tape, and the SPE was treated with poly(ethyleneimine) to achieve a cationic surface finish. Furthermore, the electrodes were alternately dipped in the poly(3,4-ethylene dioxythiophene): poly(styrene sulfonate) (PEDOT: PSS) solution and the graphene/CTS solution, depositing successive layers onto the electrode surface (Figure 6).

As a result of this modification, high selectivity and sensitivity were achieved in the detection of biomarkers, such as dopamine. The simplified hardware design and the use of layer-by-layer self-assembly technology for electrode modification represent significant advantages in device performance. The smartphone application integrates multiple detection methods, allowing for real-time data collection and analysis. This new approach, which involves the exploitation of CTS properties in addition to electrochemical techniques and smartphone applications, provides an accessible and user-friendly solution for electrochemical testing at the user level, regardless of location and time [95].

The integration of CTS and conducting polymers in the fabrication of electrochemical sensors represents a significant advancement in pharmaceutical detection. The combination of these materials offers devices with high sensitivity and selectivity due to the inherent electronic and redox properties of conducting polymers and the stability and biocompatibility of CTS. The literature shows that these sensors can detect various pharmaceutical products, such as fluconazole, daclatasvir, dopamine, nitrofurantoin, ascorbic acid, and ciprofloxacin, among others. Moreover, these sensors have lower detection limits compared with conventional methods such as chromatography or spectrometry [91].

Table 3 lists some of the Electrochemical sensors based on CTS and conducting polymers

While the fabrication of conducting polymer composites with CTS has been a significant advancement, there is still room for further optimization, particularly in improving stability, reproducibility, and response time. Additionally, a broader range of pharmaceutical products, especially those with high toxicity or low solubility, still pose a challenge for detection and quantification. Achieving these objectives requires research efforts to discover and implement new strategies for the construction and optimization of these sensors. Therefore, despite substantial progress, the potential for future development in the field of electrochemical sensors based on conducting polymers and CTS deserves an ongoing investigation.

The combination of CTS and conducting polymers has shown promise in the field of sensors. However, some several limitations and challenges must be addressed before these sensors can be widely used. The stability and reproducibility of these sensors need further optimization. Although the strong intermolecular interactions within the CTS structure provide stability, further studies exploring potential enhancements are required. Improving the response time of these sensors is essential for real-time applications. A broader range of pharmaceutical products, especially those with high toxicity or low solubility, still pose a challenge for detection and quantification [60,89,90,91]. The cost and scalability of these sensors for widespread practical applications need to be considered. In addition, the integration with smartphones and other innovative technologies requires further refinement to ensure user-friendliness and accessibility [95].

### 4.4. Electrochemical Sensors Based on Molecularly Imprinted CTS

The development of electrochemical sensors based on molecularly imprinted CTS for pharmaceutical compound detection introduces an innovative approach with impressive attributes of sensitivity and selectivity. However, the field is not without its challenges and limitations, which must be critically evaluated.

Molecular imprinting is a technique that involves designing a polymer matrix imprinted with a template molecule, thus providing a recognition component that exhibits high selectivity for the target molecule. The process and applications are detailed in the following sections, but it is essential to recognize the transformative potential of this technology while also acknowledging its current limitations.

The development of electrochemical sensors based on molecularly imprinted CTS for pharmaceutical compound detection introduces an innovative approach with impressive attributes of sensitivity and selectivity. Molecular imprinting is a technique that involves designing a polymer matrix imprinted with a template molecule, thus providing a recognition component that exhibits high selectivity for the target molecule [99]. Molecularly imprinted polymers (MIPs) have been recognized as an efficient alternative in various applications due to their remarkable properties and feasibility of use. MIPs are considered synthetic receptors obtained by using a template, a functional monomer, and a cross-linking agent. In the initial stage, the functional monomer interacts with the template, forming a “pre-polymerization complex.” By copolymerizing this complex with the cross-linking agent, an interconnected polymer is obtained. After polymerization, the functional groups of the polymer create a shell with specific recognition sites. In the final step, the target molecule is removed from the polymer through electrochemical or chemical methods, aiming to disrupt the interactions between the template and monomers [100]. Essentially, MIPs are designed to have a shape, size, and functional groups that match the template molecule, thereby enabling specific molecular recognition.

**Figure 7:**
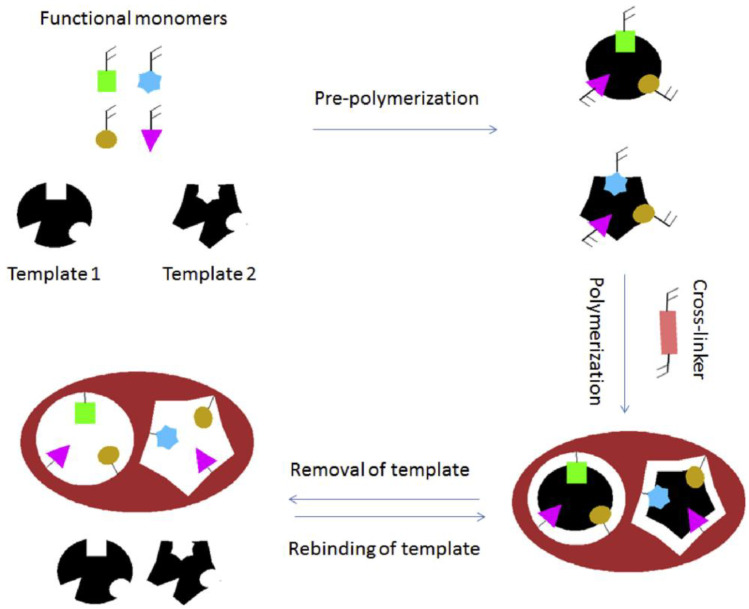
Schematic of MIP for two different templates. Step 1: Pre-polymerization. Step 2: Polymerization and cross-linking. Step 3: Removal of the template. Step 4: Recognition [99].

Figure 7 shows a schematic of MIP for two different templates.

CTS is a suitable choice for MIPs due to its functional groups that can be easily chemically modified and its solubility in aqueous solutions. A significant advantage of CTS is its ability to protect the activity of biological molecules. The properties of CTS become attractive for the preparation of template-molecule-polymer complexes. However, the choice of monomers and the challenges associated with imprinting larger structures remain areas that require further exploration and innovation [99].

The commonly used monomers in MIP synthesis, especially vinyl and acrylic monomers [101], are not soluble in aqueous solutions. This characteristic can be challenging for certain template molecules, such as peptides and proteins. For this reason, CTS represents a suitable alternative, thanks to its natural origin and wide compatibility.

Electrodeposition is a simple, easy, rapid, and cost-effective technique that allows for the controlled deposition of highly porous CTS films. The imprinting of small molecules, such as pesticides or some pharmaceutical substances, is commonly addressed for MIP synthesis and considered routine. However, the imprinting of much larger structures remains a challenge. The behavior of CTS depends on several parameters that undoubtedly influence the sensitivity of the developed sensors. The choice of functional monomer in MIP synthesis is crucial, and the development of new strategies for MIP construction continues to be an area of active research [102].

The choice of functional monomer in MIP synthesis is crucial, considering its ability to provide complementary interactions with the matrix molecules [103]. CTS has an affinity for various compounds, and the possibility of complexing the template or making chemical modifications through different strategies (non-covalent, semi-covalent, and covalent) can be easily exploited for MIP construction [104].

Several types of sensors have been developed using MIPs, including electrochemical, colorimetric, fluorescent, etc. Given the prevalence of electrochemical sensors in the market due to their simplicity, sensitivity, ease of implementation, and low measurement costs, this section will focus on the development of electrochemical sensors based on molecularly imprinted CTS (CS-MIPs). Numerous successful examples of electrochemical sensors based on CTS-MIPs for various biological molecules used as templates, such as metabolites (dopamine, L-dopa, glucose, urea), pesticides (trichlorfon, glyphosate), drugs (clenbuterol, epinephrine), and phenolic compounds (p-nitrophenol, 2,4,6-tribromophenol, bisphenol A, catechol), have been reported [91]. In general, these template molecules have a molecular weight between 100 and 200 g/mol. The common characteristic of these molecules is that they contain hydroxyl and amine groups in their structure.

Regarding the applications of CTS-based MIPs in electrochemical sensors, the work of Wu *et al.* stands out for developing a novel sensor for the detection of epinephrine using an ITO electrode modified with an imprinted CTS film containing MWCNTs and a polymerized ionic liquid. The sensor demonstrated a good linear response to the target molecule with a low detection limit [105].

Another noteworthy example is the work of Chakroun Galai *et al.*, where a CTS-based electrodepositing imprinted polymer film was developed for voltametric detection of bisphenol A (BPA). The sensor exhibited a low detection limit and excellent reproducibility [106].

Electrodeposition was also applied to an MIP-based sensor for the detection of L-dopa, using a graphene and CTS composite (GR-MIPs/GCE). The sensor construction was performed in three steps. In the first step, a deposition solution was prepared by ultrasonically dispersing graphene in a CTS solution, followed by the addition of L-dopa. In the second step, the prepared GCE electrode was immersed in the previously prepared solution, and an L-dopa-GR-CS composite film was formed by electrodeposition. In the final step, the template molecules were removed from the composite by treatment with a well-established solution of 0.1 M KCl and 100 µL of ethanol at a defined potential and time. The GR-MIPs/GCE sensor exhibited selective and sensitive detection capabilities for L-dopa, holding promise for precise applications in the pharmaceutical field and human serum analysis [107]. A similar sensor, prepared with the same nanomaterials and using the same technique, was used for the detection of L-5-hydroxytryptophan (L-5-HTP) in human serum [108].

The difference between the two sensors lay in the deposition parameters, particularly the potential and deposition time. These differences in the preparation conditions allowed for the optimization of the sensor for specific and sensitive detection of L-5-HTP in human serum, adapting it to the specific requirements and characteristics of this compound. This approach demonstrates the versatility and adaptability of the molecularly imprinted sensor construction technique, depending on the target compound and desired application.

**Figure 8:**
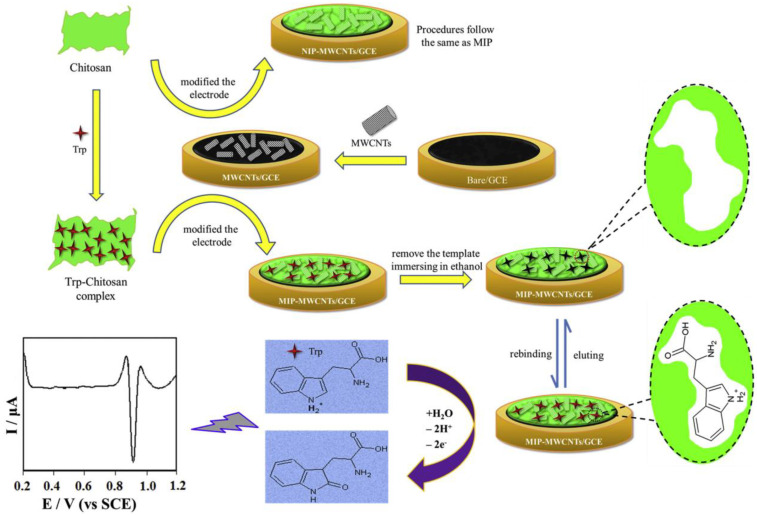
The procedure for fabrication of the MIP-MWCNTs/GCE [109].

On the other hand, Yiyong Wu’s team [109] developed an electrochemical MIP-based sensor for tryptophan detection through drop-casting, forming an imprinted CTS film with tryptophan on the surface of a previously modified GCE with MWCNTs (Figure 8).

This new method allowed for improved electron transfer and sensor selectivity towards tryptophan. The MIP-MWCNTs/GCE sensor exhibited a linear relationship between the oxidation current of tryptophan and the analyte concentrations within the established range, with a detection limit of 1.0 nM. The modified electrode demonstrated good reproducibility and stability for tryptophan detection in human serum samples [109].

Another method for preparing a sensor for bisphenol A (BPA) detection involves the use of GCE, gold nanoparticles (AuNPs), and CTS. The GCEs were polished and activated in H_2_SO_4_ solution, followed by the electrodeposition of AuNPs onto the GCE surface. Furthermore, the AuNPs were modified through self-assembly with 4-amino thiophenol (4-ATP), and the sensor was immersed in a BPA solution to allow for the formation of a molecular layer. By electropolymerization in a solution containing CTS and acetic acid, molecularly imprinted polymers (MIPs) were obtained. To complete the process, the electrodes were treated with glutaraldehyde to bind functional molecules to the polymer surface. This innovative construction method allows for the development of a high-performance sensor for BPA detection with an extended linear range and a low detection limit [110]. Although in this study, BPA is not a pharmaceutical substance and was not detected in pharmaceutical products, the newly developed sensor could be an efficient means of detecting BPA contamination in such products, contributing to ensuring the safety and quality of pharmaceuticals by identifying and eliminating potential sources of BPA contamination.

In 2019, a robust and efficient electrochemical platform for the enantioselective detection of chiral compounds was reported. Using a composite membrane of MIP and RGO on a GCE, this platform was successfully used for the detection and quantification of the S-/R-propranolol ratio, a chiral drug. The construction technique involved the selective immobilization of CTS-based MIPs and rGO on the GCE surface, ensuring precise and efficient enantioselective recognition. This electrochemical platform holds great potential for practical applications in the pharmaceutical field and clinical analysis [111].

Recently, researchers have also focused on the detection of antibiotics in various samples using molecularly imprinted polymer (MIP)-based sensors, as these bioactive compounds pose a major concern in the fields of pharmaceutical, food, and environmental analysis.

Therefore, Sandeep G. Surya *et al.* aimed to develop a biomimetic electrochemical sensor for the detection of ciprofloxacin (CIP). A GCE was modified using CTS-based gold nanoparticles (Ch-AuNPs). Using Ch-AuNPs and CIP, molecularly imprinted polymers (MIP) and non-imprinted polymers (NIP) were prepared. For the formation of the CIP-embedded MIP network, methacrylic acid, a crosslinking agent (EGDMA), and azobisisobutyronitrile (AIBN), an initiator, were used, and the pH was adjusted. In the case of the NIP, the same procedure was followed, but without adding the target molecule. Several experimental conditions influencing the sensor’s performance, such as the CIP elution agent, extraction time, volume of Ch-AuNPs applied to GCE, and equilibration time, were optimized. The Ch-AuMIP sensor exhibited high sensitivity in the concentration range of 1–100 μmol L^−1^, with a detection limit of 210 nmol L^−1^. The sensor’s applicability in detecting CIP in various real samples, such as drinking water, mineral water, milk, and pharmaceutical formulations, was studied. The developed sensor showed good selectivity for CIP, even in the presence of compounds from the same therapeutic class, such as norfloxacin and ofloxacin [112].

In the evolution of molecularly imprinted polymer (MIP)-based sensors, an innovative approach involves the use of nanocomposites derived from metal-organic frameworks (MOFs) [113] or zeolitic imidazole frameworks (ZIFs) [114]. These nanocomposites, composed of a porous carbon skeleton and metal nanoparticles or metal oxides, bring significant advancements in terms of electrocatalytic activity and electrical conductivity due to the synergy between metal and carbon. To prevent the delamination of nanocomposites, MOFs or ZIFs are grown on three-dimensional porous electrodes (3D-KSC) before calcination to form the final nanocomposites [115]. This new method ensures a uniform distribution of nanoparticles within the porous carbon structure, providing stability and superior performance for MIP sensors.

This technique was applied in 2020 [116] for the development of a molecularly imprinted sensor for dopamine (DA) detection based on Cu–Co-ZIF/CuCo_2_O_4_@porous carbon/3D-KSC nanocomposites. This new approach represented a significant innovation in the field of molecularly imprinted sensor development. By calcining Cu–Co-ZIF on 3D-KSC under a nitrogen atmosphere, the CuCo_2_O_4_@porous carbon nanocomposite was obtained, which contributed to the improved catalytic activity and stability of the sensor. The molecularly imprinted sensor was prepared by depositing a DA-CS polymeric film onto the surface of the CuCo_2_O_4_@porous carbon/3D-KSC nanocomposite through potentiostatic electrodeposition, resulting in an integrated MIPs/CuCo2O4@porous carbon/3D-KSC electrode used for DA detection. The results demonstrated that this integrated electrode exhibited higher selectivity towards DA, superior sensitivity, and improved stability [116].

Several examples of CTS-based molecularly imprinted sensors reported in the literature, along with important parameters for evaluating electrochemical performance, can be found in Table 4.

As observed from the literature analysis, electrodeposition is one of the most common and preferred methods for obtaining CTS films on conductive electrodes in the construction of electrochemical sensors. Electrodeposition can provide precise control over the thickness and composition of the CTS film but may require special equipment and a more complex process. On the other hand, drop casting can be a simple and convenient method for the rapid fabrication of sensors, but it may result in an uneven distribution of the material on the electrode surface.

The process of extracting target molecules from molecularly imprinted polymers (MIPs) is an important step, and the choice of extraction method depends on the type of template and specific application. Electrochemical sensors based on CTS offer a promising approach for the sensitive and selective detection of pharmaceutical substances, benefiting from the biocompatibility and stability provided by CTS, along with the high specificity and selectivity of biomolecules. These characteristics make these sensors efficient and cost-effective tools for monitoring and detecting pharmaceutical contamination in various environments.

In our view, the ongoing research into chitosan-based molecularly imprinted sensors is a critical step toward more efficient and accessible detection methods. However, a concerted effort to address the aforementioned limitations is essential to realizing the full potential of these sensors in practical applications.

Electrochemical sensors based on CTS offer a promising approach for the sensitive and selective detection of pharmaceutical substances. These characteristics make these sensors efficient and cost-effective tools for monitoring and detecting pharmaceutical contamination in various environments. However, a balanced view that considers both the potential and the limitations of this technology is essential for its successful implementation and further advancement.

## 5. Concluding Remarks and Perspectives

This review explores electrochemical sensors based on CTS and describes their functionality in the detection of pharmaceutical substances. CTS, a natural biopolymer, proves to be a valuable material in this field due to its beneficial properties. Its biocompatibility and stability make it an excellent material for the immobilization of a wide range of elements that can enhance its electrochemical characteristics. These features are crucial in the construction and performance of electrochemical sensors used for pharmaceutical detection.

Throughout this review, various fabrication methods of CTS-based electrochemical sensors, such as electrodeposition, drop casting, or paste formation with nanocomposites, have been carefully analyzed. Each fabrication method has its advantages and disadvantages and may be more suitable depending on the application and specific requirements of the electrochemical sensor. Additionally, pharmaceutical substances may react differently with CTS and can be influenced by fabrication methods. The chemical and physical characteristics of the pharmaceutical substance need to be considered in selecting an appropriate fabrication strategy.

In our view, the development of CTS-based electrochemical sensors represents a significant advancement in the field of pharmaceutical detection. However, it is essential to recognize that the technology is still in its nascent stages, and several challenges must be addressed to realize its full potential. The development of chemical sensors based on colorimetric test strips (CTS) has been a significant breakthrough in the field of analytical chemistry. However, there are still several challenges that need to be addressed to improve their performance. One of the most critical areas for improvement is enhancing specificity in complex sample matrices. Standardization and quality control for large-scale production are also essential for commercial success. Exploring integration with other analytical platforms can broaden the applications of CTS-based sensors. Improving the stability and reproducibility of sensor responses is vital to avoid false-positive or false-negative results. Efforts should be directed toward minimizing the detection limit, especially for substances that exist at extremely low concentrations. Improving the selectivity of sensors is necessary to avoid the influence of other potentially present compounds in the analyzed samples.

To address these challenges, several future directions can be pursued. Investigating innovative methods to enhance performance can lead to significant improvements in CTS-based sensors. Utilizing sensors in personalized medicine and other novel areas can broaden their applications further. Aligning fabrication and disposal with sustainability goals can help reduce environmental impacts. Encouraging interdisciplinary collaboration and standardization can help accelerate progress in this field. Key challenges that need to be addressed include improving the stability and reproducibility of sensor responses. Variations in sensor response can lead to false-positive or false-negative results, which can result in erroneous conclusions. Despite the limitations, the potential of CTS-based electrochemical sensors is significant. They offer a sensitive and selective approach to the detection of pharmaceutical substances, contributing to a cost-effective solution for monitoring pharmaceutical contamination in the environment. Efforts should also be directed towards minimizing the detection limit, especially for pharmaceutical substances that exist at extremely low concentrations in the environment or biological samples of patients. Finally, improving the selectivity of sensors is necessary to avoid the influence of other potentially present compounds in the analyzed samples on the sensor response.

Despite the limitations, the potential of CTS-based electrochemical sensors is significant. They offer a sensitive and selective approach to the detection of pharmaceutical substances, contributing to a cost-effective solution for monitoring pharmaceutical contamination in the environment. However, a balanced view that considers both the potential and the limitations of this technology is essential for its successful implementation and further advancement. Continued research and collaboration across disciplines will be key to overcoming the challenges and realizing the full promise of CTS-based electrochemical sensors in practical applications.

## Figures and Tables

**Table 1:** Main electrochemical sensors based on CTS and carbon nanomaterial, parameters analyzed, electrochemical technique applied, and detection limit obtained in pharmaceutical analysis.

Sensor	Analyte	Detection Technique	LOD (μmol⋅L^−1^)	Real Sample	Ref.
MWCNTs/CTS–Cu/GCE ^1^	paracetamol	DPV	0.024	human serum samples and tablet	[67]
CS–CP ^2^	paracetamol	SWV	0.508	tablets and human urines	[62]
CNT-NH_2_/CTS/GCE ^3^	mesalazine folic acid	SWV	210 52	human serum samples and pharmaceutical products	[64]
RGO-CB-CTS/GCE ^4^	dopamine paracetamol	SWV	200 53	urine samples	[69]
N-CNTs-CHIT/GCE ^5^	rabeprazole	Amp	169.2	pharmaceutical tablets	[65]
graphene–CTS/GCE ^6^	4-aminophenol	DPV	57	water samples and pharmaceutical tablet	[73]
GQD-CS/CPE ^7^	epinephrine	SWV Amp	0.0003	blood serum and in epinephrine hydrochloride injection solution	[72]
SPCB-CT/GCE ^8^	erythromycin, azithromycin, clarithromycin, roxithromycin	Amp	0.19 0.15 0.16 0.18	environmental samples and pharmaceutical samples	[74]

^1^ GCE based on MWCNTs and CTS–copper complex. ^2^ CTS-modified carbon paste electrodes. ^3^ GCE-based CTS and carbon nanotubes functionalized with amino groups. ^4^ GCE based on RGO/CB/CTS composite. ^5^ N-CNTs and CTS nanocomposite-modified GCE. ^6^ graphene–CTS composite film-modified GCE. ^7^ modified electrodes based on graphene quantum do and CTS. ^8^ Super P carbon black and CTS composite.

**Table 2:** Several electrochemical sensors based on CTS and metallic nanoparticles, along with the electrochemical technique used, the target analyte, and the corresponding detection limits.

Sensor	Analyte	Detection Technique	LOD (μmol⋅L^−1^)	Real Sample	Ref.
Aunano@CNF-CHIT/SPE ^1^	isoniazid	FIA	0.172	pharmaceutical formulations.	[84]
CdS-QDs/CS/MWCNT ^2^	warfarin	CV	0.0085	urine, serum, and milk	[85]
AgChit-CNT ^3^	clopidogrel	DPV	0.030	artificially prepared urine and pharmaceutical formulation	[80]
GCE-AgNPs-CS ^4^	doxorubicin	SWV	-	plasma	[75]
Au-In_2_O_3_-CS/ABPE ^5^	ciclopirox olamine	SWV	6.64	Batrafen cream and solution	[77]
AuNPs-GO-CTS-ECH/GCE ^6^	clindamycin	SWV	290	pharmaceutical samples	[47]
ZrO_2_@CTS/GCE ^7^	rifampicin	DPV	0.0075	serum, urine, and pharmaceutical samples	[78]
SPCE/(Cs + AuNPs) ^8^	aspirin	DPV	0.167 × 10^−9^	saliva and tablet	[76]
MWCNTs@TiO2@CS based CPE ^9^	pazufloxacin	Potentiometry	4800	human plasma	[79]
ZGC/CPE ^10^	dopamine	SWV	0.01103	pharmaceutical formulation	[83]
PtPd NPs/N-Gra/GCE ^11^	ascorbic acid, sulfite and oxalic acid	DPV	0.97 5.5 0.84	Vitamin C Injection	[81]
GO-ε-MnO_2_/CS/activated GCE ^12^	tyrosine	DPSV	0.0083	dried blood spots samples	[86]
CNHs-CHI@PtNPs/GCE ^13^	Morphine and 3,4-methylenedioxymethamphetamine	DPV	0.02 0.018	human serum and urine samples	[54]

^1^ gold nanoparticles decorated carbon nanofibers-CTS modified carbon SPCE. quantum dots onto carboxylate multiwalled carbon nanotubes and CTS composite film-modified electrode. ^2^ CdS-quantum dots onto carboxylate multiwalled carbon nanotubes/CTS composite film on the surface of a GCE. ^3^ AgNPs impregnated CTS layered carbon nanotube modified sensor. ^4^ sensor based on GCE modified with silver nanoparticles-supported poly(CTS). ^5^ nanobiocomposite Au-In_2_O_3_-CTS modified acetylene black paste electrode. ^6^ glassy carbon electrodes modified with graphene oxide and gold nanoparticles within a crosslinked CTS film. ^7^ honeycomb-like zirconium dioxide with CTS-modified GCE. ^8^ electrochemical sensors based on CTS capped with gold nanoparticles. ^9^ MWCNTs/TiO_2_/CTS Composite as a Carbon Paste Electrode. ^10^ carbon paste electrodes with ZrO_2_/graphene/CTS nanocomposite. ^11^ Pt-Pd nanoparticles/CTS/nitrogen-doped graphene-modified GCE. ^12^ Graphene Oxide-ε-MnO_2_ Microspheres/CTS Modified Activated GCE. ^13^ carbon nanohorns/CTS/Pt nanoparticles modified GCE.

**Table 3:** Electrochemical sensors based on CTS and conducting polymers, parameters analyzed, electrochemical technique applied, and detection limit obtained in pharmaceutical analysis.

Sensor	Analyte	Detection Technique	LOD (μmol⋅L^−1^)	Real Sample	Ref.
CILE\Fe_3_O_4_@PA-Ni@Pd-Cs ^1^	fluconazole	CV	80	Urine, serum, tablets	[92]
PAMAM/Graphene-CTS/GCE ^2^	rutin	DPV	0.0006	Tablet, human serum, vegetable ethanol extracts	[93]
PEDOT: PSS/CS–G/SPE ^3^	dopamine	DPV		-	[95]
PPY-CS-NPGF electrodes ^4^	metronidazole	DPV	0.0009 ^1^	pharmaceutical serum and tablet samples	[96]
CHI/VSG/PPy scaffold ^5^	dopamine	DPV	0.0194	human serum samples	[97]
Ppy-CS-Fe_3_O_4_NP/ITO ^6^	glucose	Amp	234	-	[94]
MWCNTs–CS–poly(p-ABSA)/GCE ^7^	serotonin	DPV	0.08	(spiked) human blood serum	[98]

^1^ carbon ionic liquid electrode (CILE) Fe_3_O_4_ @PA-Ni@Pd/CTS nanocomposite. ^2^ Poly(amidoamine) dendrimers/Graphene-CTS/Glass Carbon electrode. ^3^ poly(3,4-ethylene dioxythiophene), CTS, and graphene screen printed electrode. ^4^ nano-porous gold film electrodes modified with CTS/polypyrrole. ^5^ CTS/vacuum-stripped graphene/polypyrrole three-dimensional electrode. ^6^ Polypyrrole–CTS–Iron oxide (Ppy–CS–Fe_3_O_4_) nanocomposite films on ITO glass. ^7^ GCE modified with multiwalled carbon nanotubes, CTS, and poly(p-amino benzenesulfonate).

**Table 4:** Examples of molecularly imprinted CTS-based electrochemical sensors for pharmaceutical detection.

Sensor	Analyte	Detection Technique	LOD (μmol⋅L^−1^)	Real Sample	Ref.
MIP/PIL-MWNTs/ITO ^1^	epinephrine	CA	0.06	-	[105]
GR-MIP/GCE ^2^	L-5-hydroxytryptophan	DPV	0.006	human blood serum	[108]
MIP -MWCNTs/GCE ^3^	tryptophan	SDLSV ^4^	0.001	human serum samples	[109]
MIPs/AuNPs/GCE ^5^	bisphenol A	LSV	1.1 × 10^−3^	plastic and milk	[110]
MIPs/rGO/GCE ^6^	S-/R-propranolol	CV	-	-	[111]
Ch-AuMIP/GCE ^7^	ciprofloxacin	DPV	0.21	tap water, mineral water, milk, and pharmaceutical formulation	[112]
MIPs/CuCo_2_O_4_@carbon/3D-KSC ^8^	dopamine	DPV	160	injection samples spiked with different amounts of DA.	[116]
GR-MIPs/GCE ^9^	levodopa	DPV	0.012	tablet and human blood serum	[107]
MIP-CHT/GO/GCE ^10^	serotonin	DPV	1600	serum, tears, and saliva	[117]
GR–AuNPs/CS–PtNPs/gold electrode ^11^	erythromycin	CV	23	milk and honey	[118]
MIP/GCE ^12^	amlodipine	DPV	0.00000266	pharmaceutical formulations (Amlor, Mibral, Exforge)	[119]

^1^ molecularly imprinted polymer on MWCNTs pre-coated with a polymerized ionic liquid on an Indium tin oxide electrode. ^2^ graphene-CTS molecularly 4 imprinted films modified on the surface of GCE. ^3^ carbon nanotubes and molecularly imprinted polymer-modified GCE. ^4^ SDLSV-linear sweep voltammetry. ^5^ sensor based on gold nanoparticles and molecularly imprinted polymer with binary functional monomers. ^6^ a composite membrane of molecularly imprinted polymers and rGO-modified GCE. ^7^ CTS gold nanoparticles molecularly imprinted polymer. ^8^ dopamine-imprinted CTS/CuCo_2_O_4_@carbon/three-dimensional macroporous carbon integrated electrode. ^9^ A L-dopa electrochemical sensor based on a graphene-doped molecularly imprinted CTS film. ^10^ sensor based on molecularly imprinted polymer (MIP) containing CTS and graphene oxide. ^11^ gold nanoparticles fabricated molecularly imprinted polymer film at CTS–platinum nanoparticles/graphene–gold nanoparticles double nanocomposites modified electrode. ^12^ sensors based on electropolymerized molecularly imprinted poly(aniline-co-anthranilic acid).
